# DELTA^2^ guidance on choosing the target difference and undertaking and reporting the sample size calculation for a randomised controlled trial

**DOI:** 10.1136/bmj.k3750

**Published:** 2018-11-05

**Authors:** Jonathan A Cook, Steven A Julious, William Sones, Lisa V Hampson, Catherine Hewitt, Jesse A Berlin, Deborah Ashby, Richard Emsley, Dean A Fergusson, Stephen J Walters, Edward C F Wilson, Graeme MacLennan, Nigel Stallard, Joanne C Rothwell, Martin Bland, Louise Brown, Craig R Ramsay, Andrew Cook, David Armstrong, Doug Altman, Luke D Vale

**Affiliations:** 1 Centre for Statistics in Medicine, Nuffield Department of Orthopaedics, Rheumatology and Musculoskeletal Sciences, University of Oxford, Botnar Research Centre, Nuffield Orthopaedic Centre, Oxford OX3 7LD, UK; 2 Medical Statistics Group, ScHARR, University of Sheffield, Sheffield, UK; 3 Statistical Methodology and Consulting, Novartis, Basel, Switzerland; 4 Department of Mathematics and Statistics, Lancaster University, Lancaster, UK; 5 Department of Health Sciences, University of York, Heslington, York, UK; 6 Johnson & Johnson, Titusville, NJ, USA; 7 Imperial Clinical Trials Unit, School of Public Health, Imperial College London, London, UK; 8 Department of Biostatistics and Health Informatics, Institute of Psychiatry, Psychology and Neuroscience, King’s College London, London, UK; 9 Clinical Epidemiology Programme, Ottawa Hospital Research Institute, Ottawa, ON, Canada; 10 Cambridge Centre for Health Services Research and Cambridge Clinical Trials Unit, University of Cambridge, Institute of Public Health, Cambridge, UK; 11 Centre for Healthcare Randomised Trials (CHaRT), University of Aberdeen, Aberdeen, UK; 12 Warwick Medical School—Statistics and Epidemiology, University of Warwick, Coventry, UK; 13 MRC Clinical Trials Unit at University College London, Institute of Clinical Trials and Methodology, London, UK; 14 Health Services Research Unit, University of Aberdeen, Aberdeen, UK; 15 Wessex Institute, University of Southampton, Southampton, UK; 16 School of Population Health and Environmental Sciences, Faculty of Life Sciences and Medicine, King’s College London, London, UK; 17 Health Economics Group, Institute of Health and Society, Newcastle University, Newcastle upon Tyne, UK

## Abstract

Randomised controlled trials are considered to be the best method to assess comparative clinical efficacy and effectiveness, and can be a key source of data for estimating cost effectiveness. Central to the design of a randomised controlled trial is an a priori sample size calculation, which ensures that the study has a high probability of achieving its prespecified main objective. Beyond pure statistical or scientific concerns, it is ethically imperative that an appropriate number of study participants be recruited, to avoid imposing the burdens of a clinical trial on more patients than necessary. The scientific concern is satisfied and the ethical imperative is further addressed by the specification of a target difference between treatments that is considered realistic or important by one or more key stakeholder groups. The sample size calculation ensures that the trial will have the required statistical power to identify whether a difference of a particular magnitude exists. In this article, the key messages from the DELTA^2^ guidance on determining the target difference and sample size calculation for a randomised controlled trial are presented. Recommendations for the subsequent reporting of the sample size calculation are also provided.

Properly conducted, randomised controlled trials are considered to be the best method for assessing the comparative clinical efficacy and effectiveness of healthcare interventions, as well as providing a key source of data for estimating cost effectiveness.^1^ These trials are routinely used to evaluate a wide range of treatments and have been successfully used in various health and social care settings. Central to the design of a randomised controlled trial is an a priori sample size calculation, which ensures that the study has a high probability of achieving its prespecified objective.

The difference between groups used to calculate a sample size for the trial (known as the target difference) is the magnitude of difference in the outcome of interest that the randomised controlled trial is designed to reliably detect. Reassurance in this regard is typically confirmed by having a sample size that has a sufficiently high level of statistical power (typically 80% or 90%) for detecting a difference as big as the target difference, while setting the statistical significance at the level planned for the statistical analysis (usually at the two sided 5% level). A comprehensive methodological review conducted by the original DELTA (Difference ELicitation in TriAls) group^2^ ^3^ highlighted the available methods and limitations in current practice. It showed that despite the many different approaches available, some are used only rarely in practice.^4^ The initial DELTA guidance did not fully meet the needs of funders and researchers. The DELTA^2^ project, commissioned by the United Kingdom’s Medical Research Council/National Institute for Health Research Methodology Research Programme and described here, aimed to produce updated guidance for researchers and funders on specifying and reporting the target difference (the effect size) in the sample size calculation of a randomised controlled trial. In this article, we summarise the process of developing the new guidance, as well as the relevant considerations, key messages, and recommendations for researchers determining and reporting sample size calculations for randomised controlled trials (box 1 and table 1).

Box 1 **DELTA^2^ recommendations for researchers undertaking a sample size calculation and choosing the target difference**

- Begin by searching for relevant literature to inform the specification of the target difference. Relevant literature can:
  - relate to a candidate primary outcome or the comparison of interest, and;
  - inform what is an important or realistic difference for that outcome, comparison, and population.
- Candidate primary outcomes should be considered in turn, and the corresponding sample size explored. Where multiple candidate outcomes are considered, the choice of the primary outcome and target difference should be based on consideration of the views of relevant stakeholder groups (eg, patients), as well as the practicality of undertaking such a study with the required sample size. The choice should not be based solely on which outcome yields the minimum sample size. Ideally, the final sample size will be sufficient for all key outcomes, although this is not always practical.
- The importance of observing a particular magnitude of a difference in an outcome, with the exception of mortality and other serious adverse events, cannot be presumed to be self evident. Therefore, the target difference for all other outcomes needs additional justification to infer importance to a stakeholder group.
- The target difference for a definitive trial (eg, phase III) should be one considered to be important to at least one key stakeholder group.
- The target difference does not necessarily have to be the minimum value that would be considered important if a larger difference is considered a realistic possibility or would be necessary to alter practice.
- Where additional research is needed to inform what would be an important difference, the anchor and opinion seeking methods are to be favoured. The distribution method should not be used. Specifying the target difference based solely on a standardised effect size approach should be considered a last resort, although it may be helpful as a secondary approach.
- Where additional research is needed to inform what would be a realistic difference, the opinion seeking and the review of the evidence base methods are recommended. Pilot trials are typically too small to inform what would be a realistic difference and primarily address other aspects of trial design and conduct.
- Use existing studies to inform the value of key nuisance parameters that are part of the sample size calculation. For example, a pilot trial can be used to inform the choice of the standard deviation value for a continuous outcome and the control group proportion for a binary outcome, along with other relevant inputs such as the amount of missing outcome data.
- Sensitivity analyses, which consider the effect of uncertainty around key inputs (eg, the target difference and the control group proportion for a binary outcome) used in the sample size calculation, should be carried out.
- Specification of the sample size calculation, including the target difference, should be reported according to the guidance for reporting items (see table 1) when preparing key trial documents (grant applications, protocols, and result manuscripts).

**Table 1:** DELTA^2^ recommended reporting items for the sample size calculation of a randomised controlled trial with a superiority question

Recommended reporting items	Page and line numbers where item is reported
**Core items**	
(1) Primary outcome (and any other outcome on which the calculation is based)	
If a primary outcome is not used as the basis for the sample size calculation, state why	
(2) Statistical significance level and power	
(3) Express the target difference according to outcome type	
(a) Binary—state the target difference as an absolute or relative effect (or both), along with the intervention and control group proportions. If both an absolute and a relative difference are provided, clarify if either takes primacy in terms of the sample size calculation	
(b) Continuous—state the target mean difference on the natural scale, common standard deviation, and standardised effect size (mean difference divided by the standard deviation)	
(c) Time-to-event—state the target difference as an absolute or relative difference (or both); provide the control group event proportion, planned length of follow-up, intervention and control group survival distributions, and accrual time (if assumptions regarding them are made). If both an absolute and relative difference are provided for a particular time point, clarify if either takes primacy in terms of the sample size calculation	
(4) Allocation ratio	
If an unequal ratio is used, the reason for this should be stated	
(5) Sample size based on the assumptions as per above	
(a) Reference the formula/sample size calculation approach, if standard binary, continuous, or survival outcome formulas are not used. For a time-to-event outcome, the number of events required should be stated	
(b) If any adjustments (eg, allowance for loss to follow-up, multiple testing) that alter the required sample size are incorporated, they should also be specified, referenced, and justified along with the final sample size	
(c) For alternative designs, additional input should be stated and justified. For example, for a cluster randomised controlled trial (or an individually randomised controlled trial with clustering), state the average cluster size and intracluster correlation coefficient(s). Variability in cluster size should be considered and, if necessary, the coefficient of variation should be incorporated into the sample size calculation. Justification for the values chosen should be given	
(d) Provide details of any assessment of the sensitivity of the sample size to the inputs used	
**Additional items for grant application and trial protocol**	
(6) Underlying basis used for specifying the target difference (an important or realistic difference)	
(7) Explain the choice of target difference—specify and reference any formal method used or relevant previous research	
**Additional item for trial results paper**	
(8) Reference the trial protocol	

This set of reporting items has been developed with the conventional statistical (Neyman-Pearson) approach to a sample size calculation in mind. Some of the reporting items would differ if another approach were to be used. This table can be downloaded as a separate document in the web appendix; page numbers can be added electronically to the PDF document.

**Summary points**

Central to the design of a randomised controlled trial is an a priori sample size calculation, which ensures a high probability of the study achieving its prespecified main objective
An incorrect sample size can result in a study that is unable to inform clinical practice (hence directly or indirectly harming patients), or could expose excess patients to the uncertainty inherent in a clinical trial
The target difference between treatments that is considered realistic or important by one or more key stakeholder groups plays a critical part in the sample size calculation of a randomised controlled trial
Guidance on how to choose the target difference and undertake a sample size calculation for funders and researchers is presented in this article
10 recommendations are made regarding choosing the target difference and undertaking a sample size calculation, along with recommended reporting items for trial proposal, protocols, and results papers
This article on choosing the target difference for a randomised controlled trial and undertaking and reporting the sample size calculation has been dual published in *The BMJ* and *BMC Trials* journals

## Development of the DELTA^2^ guidance

**Fig 1:**
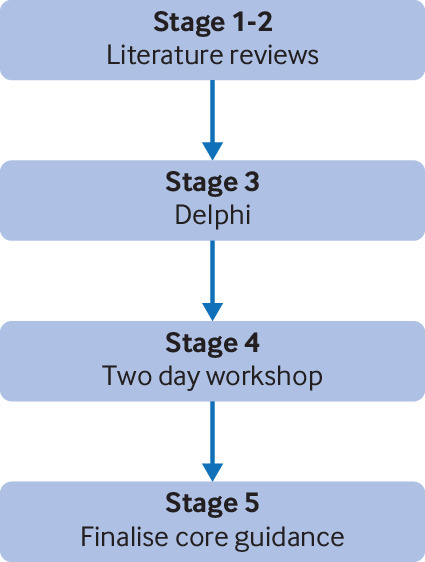
DELTA^2^ project components of work

The DELTA^2^ guidance is the culmination of a five stage process to meet the stated project objectives (fig 1), which included two literature reviews of existing funder guidance and recent methodological literature, a Delphi process to engage with a wider group of stakeholders, a two day workshop, and finalisation of the core guidance.

The literature review was conducted between April and December 2016 (searching up to April 2016). The Delphi study had two rounds: one held in 2016 before a two day workshop in Oxford (September 2016), and another between August and November 2017. The general structure of the guidance was devised at the workshop. It was substantially revised on the basis of feedback from stakeholders received through the Delphi study. In addition, stakeholder engagement events were held at various meetings throughout the development of the guidance: the Society for Clinical Trials meeting and Statisticians in the Pharmaceutical Industry conferences both held in May 2017, a Joint Statistical Meeting in August 2017, and a Royal Statistical Society Reading local group meeting in September 2017. These interactive sessions provided feedback on the scope (in 2016) and then draft guidance (in 2017). The core guidance was provisionally finalised in October 2017 and reviewed by the funders’ representatives for comment (Methodology Research Programme advisory group). The guidance was further revised and finalised in February 2018. The full guidance document incorporating case studies and relevant appendices is available here.^5^ Further details on the findings of the Delphi study and the wider engagement with stakeholders are reported elsewhere.^6^ The guidance and key messages are summarised in the remainder of this paper.

## The target difference and sample size calculations in randomised controlled trials

The role of the sample size calculation is to determine how many patients are required for the planned analysis of the primary outcome to be informative. It is typically achieved by specifying a target difference for the key (primary) outcome that can be reliably detected and the required sample size calculated. In this summary paper, we restrict considerations to the most common trial design looking at a superiority question (one which assumes no difference between treatments and looks for a difference), although the full guidance considers equivalence and non-inferiority designs that invert the hypothesis and how the use of the target difference differs for such designs.^5^

The precise research question that the trial is primarily set up to answer will determine what needs to be estimated in the planned primary analysis, which is known formally as the “estimand.” A key part of characterising the research question is choosing the primary outcome, which needs careful consideration. The target difference should be a difference that is appropriate for that estimand.^7^ ^8^ ^9^ ^10^ Typically (for superiority trials), an intention to treat or treatment policy estimand—that is, according to the randomised groups irrespective of subsequent compliance with the treatment allocation—is used. Other analyses that deal with different estimands^8^ ^9^ ^11^ of interest (eg, those based on the effect on receipt of treatment and the absence of non-compliance) could also inform the choice of sample size. Different stakeholders can have somewhat differing perspectives on the appropriate target difference.^12^ However, a key principle is that the target difference should be viewed as important by at least one (and preferably more) key stakeholder groups—that is, patients, health professionals, regulatory agencies, and healthcare funders. In practice, the target difference is not always formally considered and in many cases appears, at least from trial reports, to be determined on convenience, the research budget, or some other informal basis.^13^ The target difference can be expressed as an absolute difference (eg, mean difference or difference in proportions) or a relative difference (eg, hazard or risk ratio), and is also often referred to, rather imprecisely, as the trial “effect size.”

Statistical calculation of the sample size is far from an exact science.^14^ Firstly, investigators typically make assumptions that are a simplification of the anticipated analysis. For example, the impact of adjusting for baseline factors is difficult to quantify upfront, and even though the analysis is intended to be an adjusted one (such as when randomisation has been stratified or minimised),^15^ the sample size calculation is often conducted on the basis of an unadjusted analysis. Secondly, the calculated sample size can be sensitive to the assumptions made in the calculations such that a small change in one of the assumptions can lead to substantial change in the calculated sample size. Often a simple formula can be used to calculate the required sample size. The formula varies according to the type of outcome, how the target difference is expressed (eg, a risk ratio versus a difference in proportions), and somewhat implicitly, the design of the trial and the planned analysis. Typically, a sample size formula can be used to calculate the required number of observations in the analysis set, which varies depending on the outcome and the intended analysis. In some situations, ensuring the sample size is sufficient for more than one planned analysis may be appropriate.

When deciding on the sample size for a randomised controlled trial, it is necessary for researchers to balance the risk of incorrectly concluding that there is a difference when no actual difference between the treatments exists, with the risk of failing to identify a meaningful treatment difference when the treatments do differ. Under the conventional approach, referred to as the statistical hypothesis testing framework,^16^ the probabilities of these two errors are controlled by setting the significance level (type I error) and statistical power (1 minus type II error) at appropriate levels (typical values are two sided 5% significance and 80% or 90% power, respectively). Once these two inputs have been set, the sample size can be determined given the magnitude of the between group difference in the outcome it is desired to detect (the target difference). The calculation (reflecting the intended analysis) is conventionally done on the basis of testing for a difference of any magnitude. As a consequence, it is essential when interpreting the analysis of a trial to consider the uncertainty in the estimate, which is reflected in the confidence interval. A key question of interest is what magnitude of difference can be ruled out. The expected (predicted) width of the confidence interval can be determined for a given target difference and sample size calculation, which is another helpful aid in making an informed choice about this part of a trial’s design.^17^ Other statistical and economic approaches to calculating the sample size have been proposed, such as precision and bayesian based approaches^16^ ^18^ ^19^ ^20^ and the value of information analysis,^21^ although they are not at present commonly applied.^22^

The required sample size is very sensitive to the target difference. Under the conventional approach, halving the target difference quadruples the sample size for a two arm, 1:1, parallel group superiority trial with a continuous outcome.^23^ Appropriate sample size formulas vary depending on the proposed trial design and statistical analysis, although the overall approach is consistent. In more complex scenarios, simulations can be used but the same general principles hold. It is prudent to undertake sensitivity calculations to assess the potential effect of misspecification of key assumptions (such as the control response rate for a binary outcome or the anticipated variance of a continuous outcome).

The sample size calculation and the target difference, if well specified, help provide reassurance that the trial is likely to detect a difference at least as large as the target difference in terms of comparing the primary outcome between treatments. Failure to clarify sufficiently what is important and realistic at the design stage can lead to subsequent sample size revisions, or an unnecessarily inconclusive trial due to lack of statistical precision or ambiguous interpretation of the findings.^24^ ^25^ When specifying the target difference with a definitive trial in mind, the following guidance should be considered.

## Specifying the target difference for a randomised controlled trial

Different statistical approaches can be taken to specify the target difference and calculate the sample size but the general principles are the same. To aid those researchers new to the topic and to encourage better practice and reporting regarding the specification of the target difference for a randomised controlled trial, a series of recommendations is provided in box 1 and table 1. Seven broad types of methods can be used to justify the choice of a particular value as the target difference, which are summarised in box 2.

Box 2 **Methods that can help inform the choice of the target difference**

**Methods that inform what is an important difference**

Anchor: The outcome of interest can be anchored by using either a patient’s or health professional’s judgment to define what an important difference is. This approach can be achieved by comparing a patient’s health before and after treatment and then linking this change to participants who showed improvement or deterioration using a more familiar outcome (for which either patients or health professionals more readily agree on what amount of change constitutes an important difference). Contrasts between patients (eg, individuals with varying severity of a disease) can also be used to determine a meaningful difference.
Distribution: Approaches that determine a value based on distributional variation. A common approach is to use a value that is larger than the inherent imprecision in the measurement and therefore likely to represent a minimal level needed for a noticeable difference.
Health economic: Approaches that use the principles of economic evaluation. These approaches compare cost with health outcomes, and define a threshold value for the cost of a unit of health effect that a decision maker is willing to pay, to estimate the overall incremental net benefit of one treatment versus the comparator. A study can be powered to exclude a zero incremental net benefit at a desired statistical significance and power. A radically different approach is a (bayesian) decision-theoretic value of information analysis that compares the added value with the added cost of the marginal observation, thus avoiding the need to specify a target difference.
Standardised effect size: The magnitude of the effect on a standardised scale defines the value of the difference. For a continuous outcome, the standardised difference can be used (most commonly expressed as Cohen’s d effect size, the mean difference divided by the standard deviation). Cohen’s cutoff sizes of 0.2, 0.5, and 0.8 are often used for small, medium, and large effects, respectively. Thus, a medium effect corresponds simply to a difference in the outcome of 0.5 standard deviations. When measuring a binary or survival (time-to-event) outcome, alternative metrics (eg, an odds, risk, or hazard ratio) can be used in a similar manner, although no widely recognised cutoff points exist. Cohen’s cutoff points approximate odds ratios of 1.44, 2.48, and 4.27, respectively.^26^ Corresponding risk ratio values vary according to the control group event proportion.

**Methods that inform what is a realistic difference**

Pilot study: A pilot (or preliminary) study may be carried out if there is little evidence, or even experience, to guide expectations and determine an appropriate target difference for the trial. Similarly, a phase 2 study could be used to inform a phase 3 study, although this approach would need to take account of methodological differences (eg, inclusion criteria and outcomes) that should be reflected in specification of the target difference.

**Methods that inform what is an important or a realistic difference**

Opinion seeking: The target difference can be based on opinions elicited from health professionals, patients, or others. Possible approaches include forming a panel of experts, surveying the membership of a professional or patient body, or interviewing individuals. This elicitation process can be explicitly framed within a trial context.
Review of evidence base: The target difference can be derived from current evidence on the research question. Ideally, this evidence would be from a systematic review or meta-analysis of randomised controlled trials. In the absence of randomised evidence, evidence from observational studies could be used in a similar manner.

Broadly speaking, two different approaches can be taken to specify the target difference for a randomised controlled trial. A difference that is considered to be:

- Important to one or more stakeholder groups
- Realistic (plausible), based on either existing evidence, or expert opinion.

A large literature exists on defining and justifying a (clinically) important difference, particularly for quality of life outcomes.^27^ ^28^ ^29^ In a similar manner, discussions of the relevance of estimates from existing studies are also common; there are several potential pitfalls to their use, which needs careful consideration of how they should inform the choice of the target difference.^2^ It has been argued that a target difference should always be both important and realistic,^30^ which would seem particularly apt when designing a definitive (phase 3) superiority randomised controlled trial. In a sample size calculation for a randomised controlled trial, the target difference between the treatment groups strictly relates to a group level difference for the anticipated study population. However, the difference in an outcome that is important to an individual might differ from the corresponding value at the population level. More extensive consideration of the variations in approach is provided elsewhere.^2^ ^3^

## Reporting the sample size calculation

The approach taken to determine the sample size and the assumptions made should be clearly specified. This information should include all the inputs and formula or simulation results, so that it is clear what the sample size was based on. This information is critical for reporting transparency, allows the sample size calculation to be replicated, and clarifies the primary (statistical) aim of the study. Under the conventional approach with a standard trial design (1:1 allocation, two arm, parallel group, superiority design) and unadjusted statistical analysis, the core items that need to be stated are the primary outcome, the target difference appropriately specified according to the outcome type, the associated nuisance parameter (that is, a parameter that, together with the target difference, uniquely specifies the difference on the original outcome scale—eg, the event rate in the control group for a binary primary outcome), and the statistical significance and power. More complicated designs can have additional inputs that should be considered, such as the intracluster correlation for a cluster randomised design.

A set of core items should be reported in all key trial documents (grant applications, protocols, and main results papers) to ensure reproducibility and plausibility of the sample size calculation. The full list of recommended core items are given in table 1, which is an update of the previously proposed list.^31^ When the sample size calculation deviates from the conventional approach, whether by research question or statistical framework, the core reporting set can be modified to provide sufficient detail to ensure that the sample size calculation is reproducible and the rationale for choosing the target difference is transparent. However, the key principles remain the same. If the sample size is determined on the basis of a series of simulations, this method should be described in sufficient detail to provide an equivalent level of transparency and assessment. Additional items to give more explanation of the rationale should be provided if space allows (eg, in grant applications and trial protocols). Trial result publications can then reference these documents if sufficient space is not available to provide a full description.

## Discussion

Researchers are faced with a number of difficult decisions when designing a randomised controlled trial, the most important of which are the choice of trial design, primary outcome, and sample size. The sample size is largely driven by the choice of the target difference, although other aspects of sample size determination also contribute.

The DELTA^2^ guidance provides help on specifying a target difference and undertaking and reporting the sample size calculation for a randomised controlled trial. The guidance was developed in response to a growing recognition from funders, researchers, and other key stakeholders (such as patients and the respective clinical communities) of a real need for practical and accessible advice to inform a difficult decision. The new guidance document therefore aims to bridge the gap between the existing (limited) guidance and this growing need.

The key message for researchers is the need to be more explicit about the rationale and justification of the target difference when undertaking and reporting a sample size calculation. Increasing focus is being placed on the target difference in the clinical interpretation of the trial result, whether statistically significant or not. Therefore, the specification and reporting of the target difference, and other aspects of the sample size calculation, needs to be improved. 

## Web extra

Extra material supplied by authors

**Web appendix:** DELTA^2^ recommended reporting items for the sample size calculation of a randomised controlled trial with a superiority question

## Notes

Contributors: JAC and SAJ conceived of the idea and drafted the initial version of the manuscript. WS, LVH, CH, JAB, DAs, RE, DAF, SJW, ECFW, GM, NS, JCR, MB, LB, CRR, AC, DAr, DAl, and LDV contributed to the development of the guidance and commented on the draft manuscript. All authors have read and approved the final version. The corresponding author attests that all listed authors meet authorship criteria and that no others meeting the criteria have been omitted.

Ethical approval: Ethics approval for the Delphi study, which is part of the DELTA^2^ project, was sought and received from the University of Oxford’s medical sciences interdivisional research ethics committee (IDREC - R46815/RE001). Informed consent was obtained for all participants in the Delphi study.
